# Decision‐making about cervical screening in a heterogeneous sample of nonparticipants: A qualitative interview study

**DOI:** 10.1002/pon.4857

**Published:** 2018-08-31

**Authors:** Laura A.V. Marlow, Amanda J. Chorley, Lauren Rockliffe, Jo Waller

**Affiliations:** ^1^ Cancer Communication and Screening Group, Research Department of Behavioural Science and Health University College London London UK

**Keywords:** beliefs, cancer, cervical screening, decision‐making, oncology, PAPM, precaution adoption process model, qualitative, tailored, theory

## Abstract

**Objective:**

According to the precaution adoption process model, cervical screening nonparticipants represent a heterogeneous group including those who are unaware of, unengaged with, or undecided about screening, as well as intenders and decliners. We aimed to explore beliefs about cervical screening among these different types of nonparticipant.

**Methods:**

Semistructured interviews were carried out with women aged 26 to 65 years living in Britain (n = 29). Women were purposively sampled to represent different nonparticipant types. Interviews were transcribed verbatim, and data were analysed thematically using framework analysis.

**Results:**

The salience of some barriers to screening varied between different types of nonparticipant. Bad experiences were prominent in the discussions of women who had decided not to attend, while practical barriers were more prominent among *intenders*. There was also some overlap between nonparticipant types. For example, many of the *undecided* women described not wanting to go for screening, but with less certainty than *decliners*. Some *intenders* (particularly those who had not been screened before) described not really wanting to attend but feeling they ought to. Women's views on the invitation/reminder process also varied; *intenders* and *maintainers* appreciated written reminders and general practitioner (GP) prompts but *decliners* sometimes perceived these as “badgering.” Throughout the interviews, women described changing views on screening in the wider context of ageing and motherhood.

**Conclusions:**

The salience of screening barriers varies by nonparticipant type, offering possibilities for tailored interventions. However, the fluidity of women's stage of screening adoption might have implications for this approach to intervention design.

## 1 BACKGROUND

Regular cervical screening participation is associated with lower risk of cervical cancer and earlier‐stage diagnosis when the disease does occur.^1^ The NHS Cervical Screening Programme is estimated to avert almost 2000 cervical cancer deaths in England every year.^1^ Despite the programme's success, participation has declined over the last decade and age‐appropriate coverage (attending within 3.5 y for women aged 25‐49 or 5.5 y for women aged 50‐64) in 2017 was 72%.^2^ There is an urgent need to better understand women's reasons for nonparticipation, to reduce barriers to attendance in those who would like to take part, and to ensure that nonparticipants have made an informed choice not to attend.

In a recent survey^3^ based on the precaution adoption process model (PAPM),^4^ we found that among cervical screening nonparticipants, 50% intended to take part (*intenders*), 28% were not aware of the programme (*unaware*), and 15% had decided not to go (*decliners*). A smaller minority were *unengaged* with the decision, and very few were *undecided* about screening (for a detailed description of the PAPM, see Marlow et al^3^). In terms of psychological differences, unaware women were the most fatalistic, unengaged were most likely to avoid health information, and decliners had the lowest perceived risk of cervical cancer.^5^

Better understanding women in these different nonparticipant groups is essential for the development of appropriate interventions to increase informed screening uptake. Collecting qualitative data can contribute to a deeper understanding of relevant attributes and processes that are participant‐driven, rather than questionnaire‐driven. We therefore aimed to build on our quantitative findings using semistructured interviews to explore the beliefs and experiences of women who were purposively sampled to represent nonparticipant types described in the PAPM. While there have been many qualitative studies of cervical screening nonattenders (see review^6^), none of these have focused specifically on exploring variation across different nonparticipant groups.

## 2 METHODS

### 2.1 Participants

All women in Britain who are registered with a general practitioner (GP) are sent a screening invitation every 3 years (for those aged 25‐49) or 5 years (for those aged 50‐64 years) as part of national call‐recall programmes.^7^, ^8^, ^9^ They are invited to make a screening appointment, usually at their GP surgery. We aimed to recruit 10 women from each of the four largest nonparticipant groups identified by our survey (unaware, unengaged, decliners, and intenders).^3^, ^4^ Women were eligible to participate if they were 25‐64, lived in Britain, and had not had a diagnosis of cervical cancer or a hysterectomy.

Initially, women who had participated in our survey^3^ and given consent to be recontacted were invited to take part. We recruited further participants through posts on social media and online communities and through community groups. Online advertisements directed women to a survey about screening. Recruitment materials described the study as being about general health behaviours to avoid deterring women who had never heard of or never had cervical screening. All participants were offered a £20 voucher. Ethical approval was obtained from University College London Ethics Committee (reference: 7585/002).

We encountered two challenges with recruitment. Firstly, survey responses about screening participation were not always consistent with more in‐depth descriptions during the interviews. Thus, we included some women who were, in fact, up to date with screening and intending to go in the future (referred to as *maintainers*). In addition, recruiting women who were unaware or unengaged proved difficult despite an array of different strategies (described elsewhere^10^), and after 18 months, we ceased data collection, despite low numbers in these groups.

### 2.2 Procedure

Interviews took place between August 2016 and May 2017. Participants were given a choice of face‐to‐face or phone interviews. One dyad‐interview was carried out with two women at their request. Interviews were semistructured and followed a topic guide. We were interested in how cancer and screening fit within women's broader health concerns, to gain a more grounded account of nonparticipation. The topic guide was designed to begin with general health behaviours and experience of health services, before moving on to discuss cervical screening. Interviews were conducted by A.C. in English, recorded and transcribed verbatim. An Urdu‐speaking translator was present at three interviews. Socio‐demographic information (age, ethnicity, and screening history) was collected before or during the interview. Written informed consent was obtained.

### 2.3 Analysis

Initial free coding of 10 transcripts was carried out by A.C. and L.R. Following discussion, these codes formed an initial coding framework. A further 10 transcripts were coded by A.C. and L.R., and the framework was refined. This and all subsequent coding was carried out in NVivo version 11 (QSR International). The final framework was used to code the remaining transcripts and to recode the initial 10. A.C. then organised and summarised the data that was reviewed by L.M. Higher‐order themes were identified, and patterns within and between nonparticipant groups were examined. Since there were only a few women who were unaware/unengaged, we have sometimes described these, together with the undecided women, as those in the “earlier stages” of screening adoption.

## 3 RESULTS

Interviews were conducted with 29 women aged 25‐65 years (see Table 1 for demographic details). Two women were 65 years old and therefore no longer eligible for screening. Since the interviews were underway by the time their correct age was ascertained, we proceeded and included their data in analyses. Seven women were in the earlier stages of the PAPM (unaware, unengaged, or undecided), 16 were decliners*,* seven were intenders, and six were maintainers. The thematic structure is presented in Box S1. Supporting quotes are accompanied by a denotation of participant number and screening nonparticipant type. Additional supporting quotes are presented in Table S1.

**Table 1:** Participant characteristics

	All women (N = 29)
Age	
25‐34	5
35‐44	11
45‐54	3
55‐64	8
65+	2
Ethnicity	
White British/Irish	19
White other	1
Asian, Asian‐British	5
Black British, African, Caribbean	4
Nonparticipant type	
Unaware	1
Unengaged	1
Undecided	5
Decliner	9
Intender	6
Maintainer	5
N/A (over 65 y)	2

### 3.1 General health engagement

#### 3.1.1 Staying healthy

For all women, conceptions of a healthy lifestyle centred on diet and physical activity. A few participants discussed mental and emotional well‐being. In general, women who were decliners or maintainers described feeling healthy and portrayed a sense of being in control of their health. Despite the desire to be healthy, many women, particularly intenders, described how this was not always possible. Common barriers to healthy behaviours included being too busy with work, childcare, or other responsibilities. Existing physical or mental health conditions also acted as barriers to being healthy, as did being too “tired” or “stressed.” Women who were unengaged or undecided about screening were more varied in their discussions around health. Some did not think about their health much; others were interested in their health and discussed conscious efforts to be healthy which were similar to decliners/maintainers.

#### 3.1.2 Contact with health services

Most women did not have much contact with their GP. Even when they felt unwell, many described avoiding their GP unless they felt it was an “emergency situation” or they were “at death's door.” Women described two broad reasons for avoiding contact with primary health care unless absolutely necessary. Firstly, there was a general desire to avoid overburdening the National Health Service. Secondly, previous experiences meant some women anticipated a negative interaction that they wished to avoid. A few women did have regular contact with health services, due to preexisting conditions. These conditions could act as a barrier to healthy living on a day‐to‐day basis but appeared to facilitate health care use, giving women the opportunity to build relationships with their GP through regular contact.

While some participants mentioned vaccinations and older participants mentioned breast screening, for the majority of participants, maintaining health was based on regular lifestyle factors. Cervical screening was rarely mentioned unprompted.

### 3.2 Cervical screening

#### 3.2.1 The value of screening

A few women in the “earlier stages” described seeing little value in screening for cancer, often relating this to their perceived risk: “I'm not going to get that, so there's no point going through that process, what's the whole point?” (P10b, undecided).

Women who were intenders and maintainers generally valued screening, describing it as a good thing. Women who were decliners had mixed views on screening. Some were against screening and held strong views that it was not beneficial: “You know people can make mistakes. I could go along and they could not even pick something up. Or I could go along and be alright and then um it could develop … it could still develop between screenings” (P6, decliner). Other decliners felt screening was generally a good thing but was not something they personally wanted to participate in, because of dislike for the procedure or due to low perceived risk, or a combination of the two.

#### 3.2.2 A spectrum of experience

Although most maintainers and intenders (who had been screened before) felt screening was uncomfortable, they generally described positive experiences. These were characterised by being offered privacy, feeling secure and feeling the sample taker had been considerate and supportive: “I think it depends on who's doing your smear, every time I've had a nurse, they talk you into different conversation, before you know it, it's over” (P10a, maintainer).

Conversely, many of the decliners described negative experiences of screening, sometimes even describing it as traumatic. The emotional experience frequently appeared to be just as important as the physical aspects of the test, and the sample taker was a vital part of this. Negative previous experiences often focused on extreme pain: “I haven't been for, I'm trying to think when the last one, probably seven years ago. And for one reason only, it hurt so much I thought, I was screaming and I'm not bloody well doing that again, it was totally painful.” (P9, decliner). A number of the women described the test as “invasive” and “violating,” with one participant describing the steps she would take after attending screening to remove all traces of it. A few women described feeling rushed and not being given enough time to adjust to what for them could be a highly emotional experience. For some women, the sample taker's supportive approach was not enough to overcome their extremely negative physical experiences.

To a lesser extent, many of the unengaged and undecided women, as well as some intenders who had not been for screening before, disliked the idea of the screening procedure. Since these women had never been screened, this was based on their expectations rather than experience: “You hear the stories of other women who go through them and they're uncomfortable, and they're painful … it didn't seem like a great idea to go and have one” (P23, undecided). This imagined experience was informed by the accounts of others and descriptions of the procedure including terms such as “scrape.” Women thought that nothing could adequately prepare you for screening, and so described the “fear of the unknown.”

#### 3.2.3 Balancing the value of screening with thoughts about the procedure

Most of the maintainers and some of the intenders described screening as uncomfortable but felt it was “not a big deal” or “doesn't bother me.” For these women, the benefits of prevention, early detection, or reassurance easily outweighed the costs of screening. For some women who were undecided or decliners*,* experiences such as pain or lack of dignity meant the costs outweighed the benefits. One participant described how she had experienced abuse in the past, and so the fear of going for screening was too overwhelming to even consider, regardless of any benefits.

Feeling at low risk of cervical cancer sometimes played a role in balancing the decision to attend. For women who perceived themselves to be at low risk of cervical cancer (because they had not recently been sexually active or had only one sexual partner), the benefits of the test were less likely to outweigh the costs: “I know that this probably is a bit stupid but I haven't been sexually active for a long time and I'm aware that's one of the main risks. So I guess in my head I'm going, oh it'll be OK.” (P22, decliner). This was also the case for one women who had received the human papillomavirus (HPV) vaccination. Some of the decliners were more broadly negative about how “the system treats women” in general.

#### 3.2.4 Opportunity and capability for screening

##### Invitations and reminders

The infrequency of invitations meant it was easy to forget about screening, especially when other things were going on in the participants' lives. Some women described how the invitation had arrived at a time that clashed with other temporary occurrences such as menstruation, pregnancy, or a house move. This meant appointments were never made or were cancelled and screening was forgotten about. These discussions were dominated by intenders*:* “So when the letter came it was one of those things that I thought, OK yeah, I've got to do that and then it just got put away and every now and then I'd come across the letter and think, oh yeah, yeah I've got to do that and then we moved … and it just hasn't happened.” (P20, intender).

Most women appreciated the invitations as they acted as a reminder, but some women who were undecided or decliners described not reading invitation letters: “I didn't read it to be honest with you. I think I just threw it in the bin.” (P22; decliner). Several of the decliners were particularly negative about the letters: “I refer to them as the threatening letters.” (P27, decliner). A few decliners also described being prompted to be screened by their GP and felt their decisions not to attend were not respected.

##### Organising appointments

The practical barriers to booking a cervical screening appointment described by intenders included work or caring responsibilities or other health conditions taking priority: “Because I had a little one, so it was never enough time to do things or before that I was pregnant and before that I can't remember I was busy working probably, or it's in your mind and it's the wrong time of the month or something else like that it's just never right time to get organised and do it.”(P28, intender).

Clinic opening hours were also described as restrictive: “I don't want to go and have it done before work because I feel like I don't really want to have to, I don't know, go into work after that. But my doctors' surgery doesn't stay open that late and so just might be tricky to get an appointment, and then I have to say, oh, can I leave work early, and it just, that's just another layer of stress” (P26, intender).

### 3.3 Shifting views in the context of broader life changes

#### 3.3.1 Ageing

Older women reflected on how an accumulation of health conditions as one ages contributes to a reappraisal of one's health and provides a greater incentive to care for it. For younger participants, this could also be in relation to an imagined future and the desire to maintain health later in life. For others, ageing resulted in an increased need for privacy.

#### 3.3.2 Motherhood

Becoming a mother seemed to increase the importance of caring for one's own health. Motherhood was also discussed as a factor that increased engagement with health care, through routine medical appointments. It also influenced the way women described the privacy of their bodies and their feelings about cervical screening. Participants commonly stated that childbirth resulted in reduced embarrassment, but for one participant who was invited soon after having a baby, this was a reason to postpone screening. Having children and especially daughters also influenced conversations about sexual and reproductive health. Caring for children and a subsequent reduction in free time also affected screening attendance.

## 4 CONCLUSIONS

To our knowledge, this is the first qualitative study in the context of an organised cervical screening programme that has taken a theory‐driven approach to recruitment, purposively sampling different types of screening nonparticipants in line with the PAPM.^4^ In addition to women who intended to be screened, or had decided not to attend, we included women who were unaware and unengaged with screening (albeit a small number) and those who were undecided about screening. The main themes identified were consistent with previous studies of barriers to cervical screening.^6^ Women discussed the perceived value (or benefits) of screening as well as their perceptions of their personal risk of cervical cancer, past experiences of screening and practical barriers. As predicted by our earlier quantitative work,^5^ we found that these barriers varied between different types of nonparticipant. Most notably, bad experiences were prominent in the discussions of women who had decided not to attend, while practical barriers were more prominent among intenders. We also found variation in women's views on the invitation/reminder process, which is an integral part of organised screening programmes. While intenders and maintainers appreciated written reminders and GP prompts, these were sometimes perceived as “badgering” by decliners.

This study goes beyond our previous work by providing a deeper understanding of women's decision‐making across the nonparticipant groups; for example, the theme “balancing the value and procedure” suggests that women take into account multiple factors when making a decision about screening. The relevance of these factors seemed to vary, with some women feeling that screening was an important procedure but with potentially high emotional costs, while others felt it was an unthreatening procedure but held little value for them because of low perceived risk. This suggests that even when targeting interventions to a specific nonparticipant group, multifaceted approaches could be most effective.

**Figure 1:**
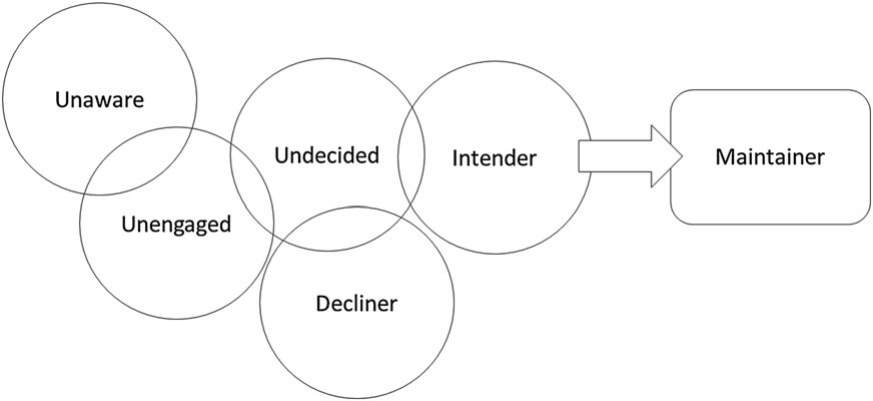
The “fuzzy” overlap of the precaution adoption process model (PAPM) in cervical screening. Unaware = have never heard of cervical screening; Unengaged = have heard of cervical screening but never thought about attending; Undecided = undecided about attending screening when next invited; Indenter = intending to go for screening but currently overdue; Decliner = decided not to attend screening in future; Maintainer = up‐to‐date with screening and planning to attend in future

Another novel finding was an apparent overlap between some of the nonparticipant types (see Figure 1). For example, many of the undecided women described not wanting to go for screening, while being less certain about this than decliners. In addition, some of the intenders (particularly those who had not been screened before), viewed screening in a way that was similar to some decliners/undecided women (not really wanting to attend, but feeling they ought to), while other intenders were more similar to maintainers (valuing screening and feeling comfortable with the procedure) with only practical barriers stopping them from participating. This “fuzzy” overlap across nonparticipant types is consistent with a qualitative study in the United States, which explored “states of nonadherence” among women overdue for mammography appointments, focusing on how well these states (that emerged from the data) reflected the stages of the PAPM. The authors concluded that “some decision levels” did not fit neatly in the PAPM and that for women who had decided not to be screened and women who intended to be screened, there were sublevels based on how certain women felt (e.g., distinguishing between definite no and qualified no: not now but I might reconsider).^11^ This “fuzziness” may also explain changes in self‐reported screening type between recruitment and interview.

The final theme, changing views on screening in the wider context of ageing and motherhood, was unprompted and arose in all interviews to varying degrees. Age and motherhood have been shown to be associated with screening attendance in quantitative studies,^3^ and our findings help to explain the mechanisms by which these factors may play a role in screening attendance. Screening information materials may need to be adapted for women at different ages and life stages.

### 4.1 Study limitations

This study has several limitations. We struggled to recruit women who were unaware of cervical screening so the conclusions predominantly reflect the views of women at the later stages of the PAPM. It is possible that different themes might have been identified had more unaware women been included; for example, quantitative work suggests fatalistic beliefs are more prominent among unaware women,^5^ but this was not widely discussed.

### 4.2 Clinical implications

There appears to be a difference in the salience of barriers to screening for women from different nonparticipant groups, and interventions could be tailored in line with this. However, there was also variation in the factors that played a part in decision‐making within groups and some overlap across groups. The fluidity of women's PAPM stage (assessed using self‐report data) has implications for the accuracy of quantitative assessment and might also have implications for the potential impact of interventions primarily aimed at specific nonparticipant types. For example, an intervention designed to reduce the intention‐behaviour gap^12^ may work for some intenders, but not those who are closer to being undecided. It is also possible that the same intervention strategy may work for women from several different nonparticipation types but for different reasons. For example, HPV self‐sampling seems to be effective at improving screening coverage in nonattenders.^13^ This strategy could address practical barriers (particularly relevant to intenders) and concerns about the screening procedure (relevant to some decliners). For women who decline screening in part because they perceive their risk of cervical cancer is low (some decliners), HPV self‐sampling may offer a low‐demand opportunity to validate this belief.

## Supporting information

Box S1: Thematic structure

Table S1: Additional supporting quotes
