# Supplementary material for: Decision‐making about cervical screening in a heterogeneous sample of nonparticipants: A qualitative interview study
Source: Psychooncology. 2018 Aug 31;27(10):2488–93. doi: 10.1002/pon.4857 (PMC6220875; doi:10.1002/pon.4857)
Supplement: Supplementary file 1 — Box S1: Thematic structure [file PON-27-2488-s001.docx]

**Supplementary Box 1: Thematic structure**

| **General health engagement**  Staying healthy  Contact with health services  **Cervical screening**  The value of screening  A spectrum of experience  Balancing the value of screening with thoughts about the procedure  Opportunity and capability for screening  *Invitations and reminders*  *Organising appointments*  **Shifting views in the context of broader life changes**  Ageing  Motherhood |
| --- |
