# Supplementary material for: Decision‐making about cervical screening in a heterogeneous sample of nonparticipants: A qualitative interview study
Source: Psychooncology. 2018 Aug 31;27(10):2488–93. doi: 10.1002/pon.4857 (PMC6220875; doi:10.1002/pon.4857)
Supplement: Supplementary file 2 — Table S1: Additional supporting quotes [file PON-27-2488-s002.docx]

**Supplementary Table 1: Additional supporting quotes**

| **General Health Engagement** | *“Um, I don’t think I’m healthy, because I have lot of pressure on my life, my husband got brain tumour, his memory going in and out. He’s lot of memory loss so I look after him and I have already two kids to look after”* [P12, unaware].  *“When I get home from work I’m really tired. Yeah I mean I’d love to go to the gym and get involved in stuff but by the time I get home from work I’m really tired”* [P18, undecided].  *“I think I’m young and healthy, there’s not very many good reasons for me to be at the doctors”* [P25, undecided]  *“She keeps a very good check on me and that every six months to make sure everything is going OK”* [P21, intender].  *“I won’t go to the doctors unless I have to. You’re just in and out and they don’t know you, they don’t have enough time. So I’ll avoid the doctors as much as I can”* [P18, undecided].  *“The NHS is such an overstretched, we don’t have the money so I think we’ve all got to do our best to look after ourselves and not take up money”* [P4, stage N/A]. |
| --- | --- |
| **Value of screening** | *“I’m not a massive believer necessarily in the screening. I don’t know, I think there’s a lot that goes on in your body all the time and I don’t know how, I don’t feel on the literature there’s enough information about the test and what it does”* [P25, undecided].  *“And I think that’s just the sort of person I am, I’d rather find out, and yes, I know it’s not the most pleasant, neither are the most pleasant things to go through, but I’ve got a lot of people to live for”* [P5, maintainer]. |
| **A spectrum of experience** | *“He told me you just going and open your legs. I say what! Why I’m open my leg and this this, I’m not doing this”* [P12, unaware].  *“Is it, is it that they use a clamp to sort of open you up a bit easier? I’m just imagining it to be very painful”* [P18, undecided].  *“It’s a little bit daunting. I think that’s the other thing, because I don’t want to book it in because then it’s a thing that I know I have to do at a certain point and because I haven’t done it before, I’m a bit worried about that, not in a rational way but just in a fear of the unknown type way”* [P26, intender].  *“Honestly all the nurses have all been lovely, they’ve all been very, did the proper, get undressed from the waist down and put a sheet over me and keep your modesty, they’re, I could never say anything about, I don’t know how they could improve the actual procedure”* [P19, intender].  *“The nurse who was, she was very nice and she was very apologetic and she was like, I’m really sorry. And I was like, OK. Obviously it wasn’t her fault and I was grateful that she apologised and acknowledged that it was painful”* [P22, decliner].  *“I would’ve liked to have gone and had the smear each time that I was called up, but I found it quite painful and I went to the one smear and I asked the nurse to stop and she wouldn’t stop. And so I had a bad experience with that, and I haven’t had one since”* [P15, decliner].  *“For me, I’ve got a history of trauma and it’s just too, I just can’t get my head round letting someone do that to me, it’s, yeah, just agreeing to being that vulnerable with someone else. I have discussed it with my doctor and to the point that I have said that I really would much rather die of cancer than that you do this, which is really extreme”* [P27, decliner]. |
| **Balancing the value of screening with thoughts about the procedure** | *“For me it just comes back to the fact that breast screening and cervical screening they’re both, they’re not very nice procedures to go through. I think that’s what puts me off more than anything to be honest”* [P2, decliner].  *“For me, I’ve got a history of trauma and it’s just too, I just can’t get my head round letting someone do that to me, it’s, yeah, just agreeing to being that vulnerable with someone else. I have discussed it with my doctor and to the point that I have said that I really would much rather die of cancer than that you do this, which is really extreme”* [P27, decliner].  *“I just think the way that we treat women’s reproductive health generally is not as advanced as it could be… And I think that if you’re going to have this procedure that is key, then I think that actually people should, someone should be working to make it as comfortable and as pain free and as comfortable as possible for women … it strikes me that we’re still doing things in the same way that we were doing them 20 year ago”* [P22, decliner].  *“Yeah I embarrassing yeah… but should go yeah because important this and it’s good idea for health”* [P3, maintainer].  *“But I know that HPV is one of, it accounts for quite a lot of the cervical cancers. And in my mind that made it OK, because I’ve had my jabs. So, I thought maybe I could get away with it”* [P23, undecided]. |
| **Opportunity and capability for screening** | *“Well I did actually get as far as making an appointment once. But then I got my period, so I had to cancel it. And then I never remade it”* [P23, undecided].  *"If you don't get that reminder then it doesn't, you know it doesn't kick in"* [P8, intender]  *“It just, it has to have it certain time of the month, has to be in the middle of your period, has to be this and that, and I just, you’re somehow always not right. So I’ve, yeah I don’t know I just think I’m not really good planning things, so I just have, I could have I suppose I could have … look at months in advance something, but I forget for today”* [P28, intender].  *“I think that fact that I’ve had another letter and it’s been pushed to the forefront of my mind again, that I need to do it”* [P26, intender].  *“I think that, when I turned 25 I think I was living in the first place I moved to in London was horrible and grotty and I just had so much going on with trying to get a decent job and working at an internship and a shop job that I just thought, oh well, I’ll deal with that later when I got the first notice letter from my GP saying, oh you should come for a smear test and I thought, yes I should but not right now and since then I’ve basically just kept letting it slide because it never feels urgent”* [P26, intender].  *“literally every time I used to go, whether it was for sciatica, migraine, the anaemia, whatever, it was brought up about smear tests … I think NHS and the doctors it’s just badger, badger, badger, have a smear test, have a smear test, have a smear test”* [P24, decliner]. |
| **Shifting views in the context of broader life changes** | *“So now I discuss a bit with my daughter because it is different in Pakistan and it is different in this country. If I don’t discuss with her, then I feel that my daughter will be confused like me”* [P12, unaware].  “*I just think as you get older, you might feel a bit, more private”* [P19, intender].  *“I think I was breastfeeding and so I thought… I don’t want people fiddling about with my bits, when I’ve still got a baby and all that… I just don’t want that just now”* [P19, intender].  *“If I walk excessively then I’ll feel it at the back of my knees which didn’t ever happen when I was young. So, my attitude to that is well yeah ok I’m ageing ok there are going to be things like that”* [P6, decliner].  “*I would like to think that I’m very aware of my body particularly as I’m getting older as well and want to do things to, I would say to promote the longevity of my body kind of thing”* [P16, decliner].  *“as soon as you give birth you’re very much in the medical... you go for all the scans erm and then there’s all the health visits afterwards … you’re in this whole world where you’re tied in with the medical profession”* [P6, decliner].  “*I always think oh my God you’ve got to look after yourself first, otherwise you can’t look after your grandchildren, you can’t look after your daughter, things like that”* [P57, maintainer].  *“It’s quite a private thing [screening] but once you’ve had kids it’s like, well stick whatever you want wherever you want you... once everybody’s seen your parts and, it’s not something that you’re very embarrassed about any more”* [P13, maintainer].  *“For me, what makes it difficult for me is childcare. I don’t literally have anybody to help me with my kids so the only time I can make an appointment to go is when the children are at nursery and school”* [P13, maintainer]. |
